# Phospholipid-Based Vesicular Systems as Carriers for the Delivery of Active Cosmeceutical Ingredients

**DOI:** 10.3390/ijms26062484

**Published:** 2025-03-11

**Authors:** Marko Lens

**Affiliations:** Leeds Institute of Medical Research, University of Leeds, Beckett Street, Leeds LS9 7TF, UK; markolens@aol.com

**Keywords:** phospholipids, liposomes, targeted delivery, cosmeceutical, nanotechnology

## Abstract

Cosmeceuticals are cosmetic products containing biologically active ingredients claiming to have drug-like benefits. In recent years, there has been a growing global demand for cosmeceuticals focusing on visible improvement of skin appearance and health. However, modern consumers are increasingly more concerned about the performance and clinical efficacy of cosmetic formulations. One of the main disadvantages of cosmeceutical preparations is the poor transdermal delivery of active ingredients included in the formulation. In response to this challenge, many phospholipid-based nanovesicular delivery systems have been developed and tested in recent years to increase the skin penetration of active cosmetic molecules. This review provides a comprehensive overview of current knowledge in the research and development of liposomal encapsulation used as delivery system in skincare and cosmeceutical products.

## 1. Introduction

Significant research and technological advancements in cosmetic chemistry, notably in the area of cosmeceuticals, have coincided with the rise in demand for high-performance skincare products and the ongoing expansion and growth of the cosmetic industry. In 1962 when receiving a medal award for his work in the field of cosmetic chemistry, Raymond Reed used a word “cosmeceutical” to define a “scientifically-designed product intended for external application that meets rigid chemical, physical and medical standards, has desirable aesthetic properties and produces useful and desired result” [1]. However, cosmeceuticals gained popularity in the 1980s as a new cosmetic category that falls between cosmetics and pharmaceuticals due to the capacity of biologically active cosmetic ingredients to influence skin health or have an impact on the skin barrier function [2]. Nowadays, cosmeceuticals are accepted as cosmetic products that exhibit pharmaceutical therapeutic advantages but not necessarily biological therapeutic benefits [3]. The effectiveness of cosmetic formulations and the verification of cosmeceutical claims are mostly dependent on the active ingredients utilised in them. Thus, when developing desirable cosmetic formulations, it is essential to evaluate their biologically active forms, mechanism of action, optimal concentration, and skin absorption [4]. In order to increase the transport of biologically active chemicals to the skin and offer their controlled and targeted release, several delivery systems have been employed. This has improved the topical efficacy of carried active ingredients and, ultimately, finished cosmetic formulations [5].

This review offers a comprehensive overview of recent developments and advances in the field of phospholipid-based delivery systems applied in cosmetic chemistry for safe and improved topical administration and efficacy of biologically active ingredients included in cosmeceutical skincare formulations.

## 2. Phospholipids

Phospholipids are a class of lipids that represent the major components of all cellular membranes. Their distinctive molecular structure consists of a hydrophilic head and two hydrophobic tails (a glycerol backbone esterified in positions *sn*-1 and *sn*-2 with fatty acids (fatty acyl molecules) and in position *sn*-3 with phosphate, further esterified with an additional alcohol) [6]. This amphiphilic nature of phospholipids allows them to spontaneously form a lipid bilayer with the hydrophilic heads facing outward, interacting with the surrounding aqueous environment and the hydrophobic tails facing inward towards each other [7]. Although cell membranes contain many different types of phospholipids, the two most prevalent forms of phospholipids found in the phospholipid mass of eukaryotic cells are phosphatidylcholine (formerly known as lecithin) and phosphatidylethanolamine [8]. While in the past it has been thought that phospholipids had only a structural role, nowadays it has been found that these active lipid molecules also play functional roles in many cellular processes, such as membrane protein regulation, membrane trafficking, cell growth, apoptosis, and intracellular signalling [9]. Phospholipids are widely used as drug carriers due to their different beneficial features, including self-assembly, amphiphilic nature, emulsifying and solubilising qualities, wetting capacity, and biocompatibility [10].

Both natural (obtained from vegetable or animal sources) and synthesised phospholipids can be employed in medicinal applications [11]. However, natural phospholipids (soybean lecithin, sunflower seed lecithin, and egg yolk lecithin) have been used broadly because they are biodegradable, allergy-free, and easily modified to promote drug bioavailability [12].

In recent years, phospholipid-based systems have been used to transport a large number of active ingredients (peptides, vitamins, botanical extracts, etc.) into the skin. The skin serves as an efficient physical barrier in respect to the environment, protecting the organism from pathogens as well as physical and chemical assaults. The outermost layer of the skin (stratum corneum), which has a thickness of 15–20 μm, acts as the responsible barrier that controls penetration and absorption of external molecules that can pass through it via three different routes: transdermal (intercellular or transcellular) and appendageal (through sweat glands or hair follicles) [13]. However, the penetration of phospholipid carriers is complex and involves different primary mechanisms: structural disruption of the stratum corneum, elastic deformation of vesicles and their squeezing through the stratum corneum, interaction with lipids from the stratum corneum, size and surface charge modulations of phospholipid vesicles, and fluidisation of lipid bilayers.

Impaired barrier function is a significant factor in the pathogenesis of many skin disorders since one of the epidermal functions is to protect against substantial water loss outside the barrier (also known as transepidermal water loss, or TEWL) [14]. The stratum corneum is composed of these lipids: 50% ceramides, 28% cholesterol, 17% free fatty acids, and 5% cholesteryl sulphate [15]. The negligible phospholipid content (<0.1%) in the stratum corneum presents a significant challenge to the use of phospholipid carriers in transdermal drug delivery. To modify the barrier properties and ultimately improve skin penetration, a number of permeation enhancers have been added to the liposome composition (cholesterol, alcohol, fatty acids, etc.).

Currently, different phospholipid-based delivery systems are available in cosmetic chemistry: liposomes, phytosomes, transfersomes, hyalurosomes, ethosomes, transethosomes, glycerosomes, invasomes, ultrasomes, and marinosomes.

## 3. Liposomes

Liposomes represent the most popular and well-developed form of phospholipid-based vesicular delivery systems used in cosmetic chemistry. The concept of liposomes was introduced in 1965 when Bangham described multilamellar phospholipid vesicles using electron microscopy and reported that these spontaneously formed lipid bilayer structures could capture cations such as potassium and sodium [16,17]. In 1968, Weismann used the term “liposomes” as an analogy to lysosomes [18]. In 1967, Papahadjopoulos produced unilamellar vesicles that due to their capacity to entrap only a relatively low volume of aqueous space per mole of lipid could not encapsulate large molecules [19]. The first example of the use of liposomes as therapeutic agents occurred in 1971 when Gregoriadis and his collaborators incorporated enzymes into liposomes for enzyme replacement therapy [20].

### 3.1. Liposomes: Structure, Classification, Preparation Methods, and Characteristics

Liposomes are artificial spherical structures (vesicles) constituted of a well-defined aqueous core (hydrophilic cavity) confined by a phospholipid bilayer. Liposomes can have one or more phospholipid bilayers, each of them separated by an aqueous layer.

**Figure 1:**
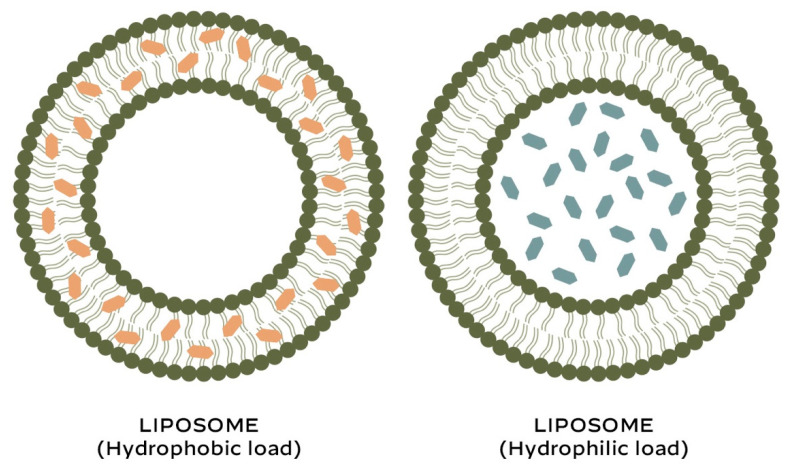
Liposomes: encapsulation of active compounds.

Due to their biphasic nature, liposomes are able to encapsulate both hydrophilic and lipophilic active molecules (Figure 1).

Based on the structural features that include lamellarity (number of bilayers) and size, Pattni et al. classified liposomes in three major categories: unilamellar (small, large, and giant), oligolamellar, and multilamellar vesicles [21] (Table 1). Later on, it has been proposed to include multivesicular liposomes as a special category to this classification.

Liposomes have also been classified into the following six categories according to their composition and application: (1) conventional liposomes, (2) charged liposomes (3) stealth stable liposomes, (4) actively targeted liposomes, (5) stimuli-responsive liposomes, and (6) bubble liposomes [22].

The most common type of liposomes are conventional liposomes, the first-generation liposomes composed of phospholipids with or without cholesterol. The purpose of adding cholesterol is to enhance the properties of lipid bilayers (improvement of vesicle stability, modulation of the membrane fluidity, increase in drug retention, regulation of permeability, and influence on size and shape of phospholipid vesicles).

Despite the fact that numerous liposome preparation methods have been reported, two main groups of conventional techniques for liposome preparation may be distinguished: active loading and passive loading (mechanical dispersion, solvent dispersion, detergent removal) [23]. Active loading methods integrate active compounds after liposome creation, whereas passive loading methods encapsulate active molecules during or prior to liposome synthesis.

Among different techniques of mechanical dispersion, sonication is the most extensively used method for the preparation of SUVs, while the thin film hydration is the most popular method for the preparation of MLVs [24,25].

However, a number of novel methods for liposome preparation have emerged recently, offering different advantages compared to widely used conventional methods (such as higher encapsulation efficiency, versatility, and scalability) [26].

Main advantages and disadvantages of liposomes are listed in Table 2 [23,25,27].

The effectiveness of liposomes in biological systems depends on the physical properties of liposomes (size, polydispersity index- PDI, Zeta potential, conductivity, and mobility) as well as their chemical, physical, and biological stability [28,29].

### 3.2. Use of Liposomes in Cosmetics

The application of liposomes in cosmetic chemistry has been extensively investigated in the past few decades. In fact, different active cosmetic molecules have been encapsulated into liposomes in order to enhance their stability and delivery to the skin and thus improve the efficacy of cosmetic formulations [30].

One of the most critical measurements in the development and manufacturing of liposomes is the encapsulation efficiency (EE%), which is the percentage of active molecule that is successfully entrapped into liposomes over the initial concentration of active molecules used for the liposome preparation. Encapsulation efficiency depends on many factors: the concentration and properties of active molecule, the size, composition, and surface charge of the liposomes, and the method used for liposome preparation [31,32].

Many botanical extracts with antioxidant activity have been loaded into liposomes for skincare application. The purpose for encapsulation of botanical extracts was not only the enhancement of their bioavailability but also protection from the oxidation to which polyphenols are particularly susceptible. Plant polyphenols demonstrate antioxidant, anti-inflammatory, and photoprotective properties. However, data from the literature indicate that the encapsulation efficiency of liposomes loaded with different plant extracts varies greatly.

Turmeric (*Curcuma longa*) extract rich in curcuminoids possesses antioxidant, anti-inflammatory, and photoprotective properties. In order to enhance the skin penetration of this extract, using lipid film hydration technique, Kaur and Sarf prepared phosphatidylcholine-cholesterol liposomes (size 210–265 nm) in which turmeric extract was loaded in three different concentrations: 0.5%, 1%, and 2% [33]. Findings from their study demonstrated that the encapsulation efficiency rose as the concentration of turmeric extract increased: 43.1% ± 1.4 at 0.5% concentration, 45.6% ± 2.2 at 1% concentration, and 46.8% ± 1.4 at 2% concentration of turmeric extract. Additionally, they showed that turmeric-loaded liposomes had superior skin penetration compared to plain extract, which led to a significant improvement in skin moisture and sebum content.

Numerous studies have demonstrated that the widely used plant gotu kola (*Centella asiatica*), rich in asiaticoside and madecassoside, possesses excellent antioxidant properties and promotes fibroblast proliferation and collagen biosynthesis. An aqueous extract of Centella asiatica was encapsulated by Kwon et al. into liposomes (115 nm in size) with an approximate 67% encapsulation efficiency [34]. The conducted study showed that liposomes at a concentration of 0.5 mg/mL significantly decreased matrix metalloproteinase (MMP-1) expression and inhibited hyaluronidase expression in UV-irradiated cells as compared to the crude plant extract. These results indicate that gotu kola-loaded liposomes can be a useful active ingredient in a cosmetic product formulated to boost collagen production and reduce the appearance of fine lines and wrinkles.

Pinsuwan et al. entrapped roselle (*Hibiscus sabdariffa*) extract into soybean-derived phosphatidylcholine liposomes (332 nm in size) achieving an encapsulation efficiency of 83% [35]. Performed testing revealed that these liposomes with antioxidant and anti-inflammatory properties have better skin permeation and reduced skin irritability in comparison to a simple extract. After being stored for two months at 4 °C with protection from light, this liposome formulation was shown to be stable.

Silymarin, a standardised extract from the fruits of milk thistle (*Silybum marianum*), has been utilised in cosmetic formulations due to its potent antioxidant, anti-inflammatory, and UV protective properties. Karkad et al. encapsulated silibinin, the major active compound of silymarin, in various phospholipid liposomes in an effort to increase its stability and bioavailability [36]. They demonstrated that the encapsulation efficiency depends on the type of liposomes: 89.7% ± 1.4 for MLVs, 74.9% ± 1.0 for SUVs, and 62.5% ± 1.9 for lyophilised liposomal vesicles. The results presented showed that the integrity of the bilayer membrane of liposomes was impaired by the process of lyophilisation due to the freeze-drying.

A rosehip (*Rosa canina*) extract rich in isoquercetin has been used as an active cosmetic ingredient due to its antioxidant and anti-ageing properties. Using the proliposome method, Jovanovic et al. created small liposomes (approximately 250 nm in size) loaded with rosehip extract, achieving a high encapsulation efficiency of 90.8% ± 0.4% [37]. The proliposome method involved stirring a rosehip extract in 70% ethanol and phosphatidylcholine at 50 °C. Water was added after cooling, and the resulting emulsion was agitated for an hour at 800 rpm. The liposomes were then subjected to sonication. Created liposomes had negative Zeta potential (from −21.3 to −22.4 mV), PDI ~0.440, and conductivity of 0.007 ± 0.001 mS/cm. Rosehip-loaded liposomes induced significantly higher inhibition of ABTS and DPPH radicals compared to pure rosehip extract (63.0% and 74.4% versus 45.4% and 57.7%, respectively).

It has been shown that the incorporation of other active ingredients into liposomes provides better bioavailability and clinical efficacy than the active ingredient alone. According to Takahashi et al., liposomes loaded with commonly used aloe vera leaf gel extract (AGE) significantly increased the collagen synthesis when compared to AGE alone (23%, versus 4%, respectively) [38]. The same study demonstrated that fibroblast proliferation was considerably higher when fibroblasts were treated with AGE-liposomes incorporating extract at 4 g/mL and 20 g/mL than with AGE alone at the same concentration (77% and 101% versus 41% and 60%, respectively).

By adding micronised propolis to large unilamellar liposomes (255 nm in size, PDI 0.355, Zeta potential −21 mV), Spanidi et al. were able to achieve an encapsulation effectiveness of 84% ± 4% [39]. At 37 °C and pH 7.2, the cumulative release of the polyphenols was 24% after 8 h. In vitro assays and tests on a reconstituted skin model showed that this controlled release delivery system exhibits antioxidant and anti-ageing potential, making it suitable for cosmetic application.

The usage of peptides, particularly those having biomimetic properties, has noticeably grown in cosmeceutical chemistry over the past ten years. One of the most explored peptides is the copper-binding tripeptide (GHK-Cu), which is made up of amino acids glycine, histidine, and lysine. GHK-Cu acts as a signal peptide that promotes wound healing and skin repair. Using the thin film hydration method, Dymek et al. prepared anionic liposomes (approximately 100 nm in size) as potential carriers for GHK-Cu, suggesting that anionic liposomes with a lipid concentration of 25 mg/cm^3^ and with 0.5 mg/cm^3^ GHK-Cu solution would guarantee the highest encapsulation efficiency of 31.7% ± 1.0 [40]. The elastase inhibition assay showed that GHK-Cu liposomes inhibit elastase activity by 48.9%, which may explain the copper tripeptide’s ability to boost collagen synthesis and reduce the appearance of wrinkles.

In order to investigate the potential use of poorly soluble fullerenes for topical applications in dermatology and cosmeceuticals, Lens et al. encapsulated fullerene C_60_ and two newly synthesised fulleropyrrolidine derivatives, namely Q-C_60_ [*N*-methyl-(2-quinolyl)fulleropyrrolidine_60_] and I-C_60_ [*N*-methyl-(2-indolyl)fulleropyrrolidine_60_] into multilamellar liposomes [41]. They demonstrated that fullerenes encapsulated in liposomes have strong antioxidant activities: C_60_, I-C_60_, and Q-C_60_ all showed similar capacities to prevent lipid peroxidation, while C_60_ exhibited a slightly higher ability to remove hydroxyl radical (•OH) than Q-C_60_ and I-C_60_.

## 4. Other Phospholipid-Based Vesicular Delivery Systems

**Figure 2:**
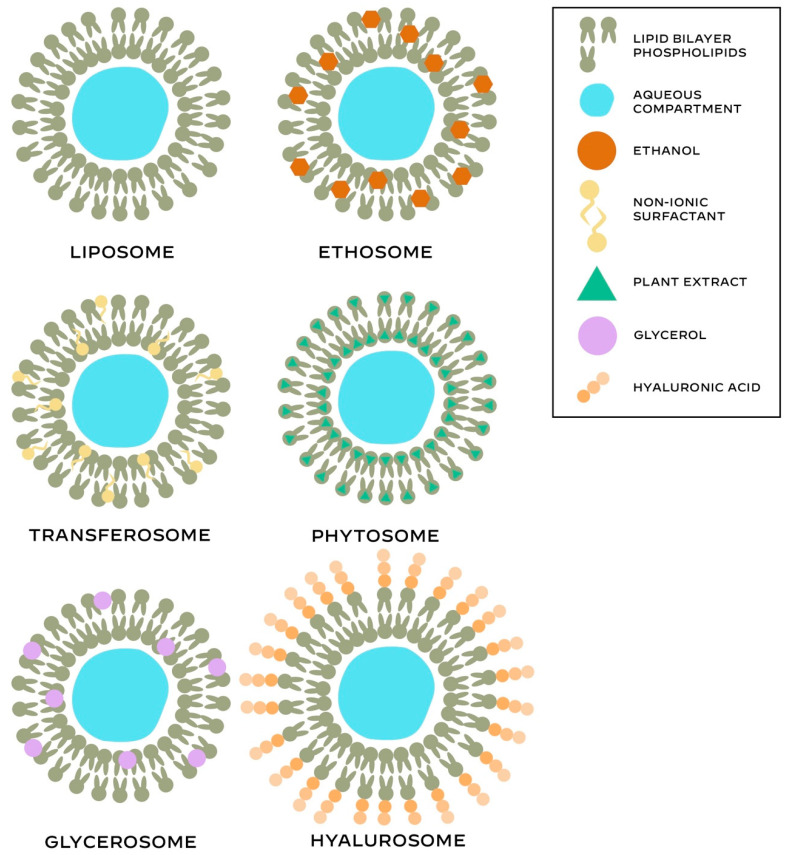
Main structural features of the different phospholipid-based systems.

Advancements in research and technology in the field of cosmetic chemistry led to the development of novel delivery systems to promote controlled and targeted delivery of various active cosmetic ingredients and enhance the efficacy of cosmeceutical formulations [42]. Different types of phospholipid-based vesicular delivery systems are presented in Figure 2.

### 4.1. Phytosomes

Phytosomes (also called herbosomes or phyto-phospholipid complexes) are vesicular delivery systems that represent complexes that combine phospholipids and a phytochemical directly incorporated into phospholipids, primarily phosphatidylcholine [43,44]. Phytosomes are structurally similar to liposomes, with the exception that they have hydrogen bonds between the hydrophilic portions of phospholipids and the phytochemicals. Phytochemicals are biologically active chemical compounds produced by plants. Two major classes of phytochemicals include carotenoids and polyphenols (phenolic acid, flavonoids, stilbenes, and lignans). Phytosomes typically range in size from 50 nm to several hundred μm. The main advantages of phytosomes over liposomes include higher encapsulation of active ingredients, better stability, and superior absorption and bioavailability [45].

Phytochemicals from various plants (18-Beta glycyrrhetinic acid from *Glycyrrhiza glabra,* ginko flavonol glycosides from *Ginkgo biloba*, epigallocatechin 3-O-gallate from *Camellia sinensis*, procyanidins from *Vitis vinifera*, silybin from *Silybum marianum*, polyphenols from *Olea europea*, terpenes from *Centella asiatica*, etc.) have been used to create phytosomes for the preparation of cosmetic formulations [43,46,47].

Black mustard (*Brassica nigra*) seeds contain a glucosinolate called sinigrin, which Mazumder et al. used to create a phytosome complex. A controlled and prolonged release of sinigrin from the phytosome complex to the stratum corneum layers of the skin greatly accelerated wound healing, according to the results of an in vitro study on human skin [48]. At a concentration of 0.14 mg/mL, the sinigrin–phytosome complex after 42 h completely healed the wound, whereas sinigrin alone only demonstrated 71% wound closure.

### 4.2. Transferosomes

Transferosomes are a special type of liposomes composed of four different components: phospholipids, an edge activator (10–25%), ethanol (usually under 10%), and water as the vehicle [49]. These phospholipid-based vesicular carriers with a typical size of less than 300 nm are ultra-deformable and elastic due to the presence of an edge activator capable of modifying the assembly of the bilayer. In transferosomes, hydrophilic active molecules are entrapped within the aqueous central cavity, while hydrophobic active molecules are nested within the phospholipid bilayer [50]. The most common edge activators used for formation of transferosomes are surfactants (Tweens and Spans), bile salts (sodium cholate and sodium deoxycholate), and dipotassium glycyrrhizinate. In comparison to liposomes, transferosomes are more stable and have better penetration. A high degree of deformability of vesicles allows transferosomes to easily penetrate the stratum corneum and reach deeper layers of the skin. Transferosomes are biocompatible and biodegradable, just as liposomes. Their highly malleable vesicles permit slow and gradual release of active compounds that are efficiently encapsulated and protected from metabolic degradation [51].

Transferosomes have been utilised in cosmetic chemistry for the delivery of herbal extracts, antioxidants, and hyaluronic acid.

To combat oxidative stress, Castangia et al. prepared transferosomes using a peel extract of a Brazilian berry fruit called jaboticaba (*Myrciaria jaboticaba*) [52]. Polyphenols, which are abundant in jaboticaba, have excellent antioxidant, anti-inflammatory, and anti-microbial properties. To increase vesicle stability, jaboticaba extract was encapsulated in transferosomes enriched with hyaluronic acid (about 91 nm in size, PDI 0.24, and Zeta potential—20 mV), achieving a high encapsulation effectiveness of 91% ± 2.0. According to in vitro assays, these transferosomes efficiently prevent the cellular damage induced by hydrogen peroxide and promote wound healing in human keratinocytes. Hyaluronan-transferosomes loaded with jaboticaba extract demonstrated 100% wound healing after 48 h of in vitro treatment of skin lesions. In contrast, 50% and 40% wound closure were observed with extract alone and no treatment, respectively.

Avadhani et al. encapsulated epigallocatechin-3-gallate (EGCG) from green tea along with hyaluronic acid (HA) in transferosomes using a modified thin film hydration method [53]. Although various formulations were evaluated, the optimised transferosomal batch containing 10 mg of EGCG and 1.75 mg of HA had a size of 101.2  ±  4.82 nm and the encapsulation efficiency value for EGCG and HA of 76.53  ±  2.68 and 48.57  ±  4.35, respectively.

According to in vitro antioxidant tests (DPPH and ABTS assays), lipid peroxidation assay, and measurements of intracellular ROS level, transferosomes loaded with EGCG and HA exhibit excellent free radical-scavenging efficacy and demonstrate strong UV-protection potential. Results evaluating MMP-2 and MMP-9 expression showed that transferosomes containing EGCG and HA inhibit degradation of type IV and type VII collagen, suggesting that transferosomes may have significant anti-wrinkle activity in cosmetic formulations.

Wu et al. used a high-pressure homogenisation process to encapsulate resveratrol in transferosomes because of its poor water solubility, instability, and low bioavailability [54]. Transferosomes loaded with resveratrol (58 nm in size, PDI 0.304, Zeta potential—0.54 mV) showed superior antioxidant capacities (DPPH and ABTS radical scavenging activity) and improved skin penetration of resveratrol following encapsulation, with a maximum entrapment efficiency of 67%. These findings suggested that resveratrol-transferosomes can be used in different cosmetic formulations targeting the mechanism of oxidative damage.

### 4.3. Hyalurosomes

Hyalurosomes are a special type of liposomes in which phospholipids are coupled with sodium hyaluronate. Comparing to liposomes due to the presence of sodium hyaluronate, hyalurosomes have larger size and higher mechanical resistance caused by the immobilising effect of hyaluron polymer [52].

Manca et al. at loaded a high concentration of curcumin into hyalurosomes [55]. These curcumin-loaded unilamellar or oligolamellar phospholipid vesicles (112–220 nm in size) demonstrated potent antioxidant and anti-inflammatory properties in in vivo experiments, which helped to reduce inflammation and oedema formation and promote extensive skin reepithelization.

The same group of researchers incorporated liquorice (*Glycyrrhiza glabra*) root extract in small hyalurosomes (≤100 nm), showing that proposed vesicular systems have significantly better efficacy in combating oxidative stress-induced skin damage and in the promotion of skin reepithelization than raw glycyrrhizin (the major active constituent of liquorice root glycyrrhizin) [56].

In order to assess their synergistic efficacy, Sklenarova et al. co-loaded oleuropein (phenolic compound from olives) and lentisk oil (oil from mastic tree—*Pistacia lentiscus*) into various types of liposomes [57]. Oleuropein is a potent antioxidant, while lentisk oil has been traditionally used to treat burns, wounds, and sores due to its excellent repairing properties. Using oleuropein (20 mg/mL), lentisk oil (75 mg/mL), and hyaluronic acid (1 mg/mL), they prepared oligo-lamellar hyalurosomes (about 150 nm in size, PDI 0.14, Zeta potential −64 mV) with an encapsulation efficiency of 90% ± 2. Multiple in vitro tests showed that hyaluron-phospholipid vesicles loaded with two phytochemicals promote fibroblast proliferation and wound healing while reducing the production of inflammatory markers MMP-1 (metalloprotease-1) and Il-6 (interleukin-6). Antioxidant, anti-inflammatory, and regenerative results achieved in this study indicate that hyalurosomes co-loaded with oleuropein and lentisk oil may have a therapeutic value in the topical treatment of different skin disorders and premature skin ageing.

Recently, Perra et al. incorporated small amounts (5–20 mg/mL) of biotechnologically produced lavender extract (*Lavandula angustifolia*) in hyalurosomes achieving encapsulation efficiency of 100% [58]. This study showed that hyalurosomes loaded with lavender extract rich in rosmarinic acid protect skin fibroblasts from oxidative stress, suggesting that these nanocarriers can be utilised in the preparation of various dermatological and cosmetic topical formulations.

### 4.4. Ethosomes

Ethosomes are modified liposomes, composed of phospholipids, high concentrations of ethanol (20–45%), and water [59]. In addition to these conventional ethosomes, there are also so-called binary ethosomes, which contain one more ingredient—another type of alcohol, usually isopropyl alcohol or propylene glycol.

As the third generation of lipid carriers, ethosomes have higher entrapment efficiency, better stability, improved penetration, and smaller size than traditional liposomes [59,60].

Sallustio et al. encapsulated an extract from rosehips (*Rosa canina*) into different phospholipid vesicles: liposomes, hyalurosomes, and ethosmes [61]. The encapsulation efficiency of ethosomes was 92.3%, which was significantly higher than the respective efficiencies of 65.5%% and 63.9% achieved for liposomes and hyalurosomes. Loaded ethosomes had vesicles that were 196 nm in size, compared to 1971 nm and 2236 nm for loaded liposomes and hyalurosomes, respectively. PDI of ethosomes was 0.20, 0.45 of liposomes and 0.32 for hyalurosomes, while their Zeta potential was −37.4 mV, −47.2 mV and −56.2%, respectively. In vitro experiments showed that while the solution containing rosehip-loaded ethosomes preserved after 6 h high antioxidant activity (80.7%), the suspension with free rosehip extract rapidly lost it antioxidant potential (15.5%) during the same time period.

Khan et al. prepared an ethosomal suspension with 2% arbutin using the cold method to assess its effectiveness in reducing skin hyperpigmentations [62]. Binary ethosomes with a size of about 196 nm and an encapsulation efficiency of 93.46% ± 2.40 included phospholipids at 2%, ethanol at 40% and propylene glycol at 20%. According to an in vivo investigation, ethosomal gel containing arbutin significantly decreased the amount of melanin. Using a stable ethosomal gel loaded with an arbutin-rich bearberry (*Arctostaphylos uva-ursi*) extract, the same research team demonstrated an encapsulation efficiency of 97.51% ± 0.023 and an in vitro skin permeation of 79.88% ± 0.013 [63]. Depigmentation efficacy was shown in in vivo studies, which is important for potential use of these ethosomes in cosmetic formulations with anti-ageing claims.

### 4.5. Transethosomes

Transethosomes are regarded as the second generation of ethosomes, since they have an edge activator (surfactants) added to the phospholipid architecture of ethosomes [52]. Being more elastic and malleable than traditional ethosomes, transethosomes are essentially a hybrid of ethosomes and transferosomes.

A study using transmission electron microscopy study demonstrated that both ethosomes and transethosomes traverse the stratum corneum through the intercellular space, pass transcellularly from stratum granulosum to the stratum basale, while in the intracellular space they undergo enzymatic degradation [64]. Findings from this study suggested that transethosomes are suitable for transdermal delivery to more superficial layers of the skin, while ethosomes can provide deeper delivery.

Transethosomes have been used as valuable nanocarriers of different drugs and active molecules in the field of dermatology and cosmetics because of their efficient transdermal delivery [65].

Basto et al. loaded niacinamide (an amide form of vitamin B3) into three different types of transethosomes—TE (TE tween 80, TE oleic acid, and TE jojoba oil) with loading capacities of 5.3%, 6.7%, and 7.6%, respectively [66]. Achieved values of Zeta potential (−17 mV for TE tween 80, −40 mV for TE oleic acid, and −21 mV for TE jojoba oil) indicated the necessity to confirm stability of prepared nanovesicles during their storage. Amount of niacinamide retained in the skin (skin deposition rate) was 9.2%, 10.9%, and 27.8% for TE tween 80, TE oleic acid, and TE jojoba, respectively. This suggested that the nanogels have the highest levels of niacinamide retention and skin permeation. In vitro tests on human keratinocytes showed that niacinamide-transethosomes provide a potent photoprotecive effect against UVB-induced skin damage.

Transethosomes (size 146 nm) showed the highest encapsulation efficiency of 97.63% and deposition of over 95%. Clinical study showed that administration of solution containing CoQ10-transethosomes has a therapeutic benefit in the treatment of androgenic alopecia.

A variety of phospholipid vesicles, including liposomes, transfersomes, ethosomes, cerosomes, and transethosomes, were used by El-Zaafarany et al. to encapsulate CoQ10 (ubiquinone) [67]. The maximum encapsulation effectiveness of 97.63% and deposition of over 95% were demonstrated by transethosomes (size 146 nm). The results from a clinical trial showed that administration of solution containing CoQ10-transethosomes has a therapeutic benefit in the treatment of androgenic alopecia.

### 4.6. Glycerosomes

Glycerosomes are modified liposomes composed of phospholipids and glycerol (20–30%) with or without cholesterol. It has been suggested that glycerosomes promote the accumulation of phosphatidylcholine in different skin layers because of the moisturising and humectant properties of the added glycerin [52].

Allaw et al. incorporated an extract of *Hypericum scruglii* (a plant from the St. John’s wort family growing in Sardinia) in glycerosomes, glu-glycerosomes (with added dextrin), gel-glycerosomes (with added gelatin), and hyal-glycerosomes (with added hyaluronan) [68]. The encapsulation efficiency was lowest for glycerosomes (85% ± 7) and highest for gel-glycerosomes (97% ± 4). Zeta potential ranged from −39 mV for gel-glycerosomes up to −53 mV for glycerosomes. Experiments conducted on human keratinocytes revealed that nanovesicles loaded with the Hypericum scruglii extract that is rich in flavonoids and phenolic compounds had a protective impact against oxidative stress and aid in wound healing. The use of glu-glycerosomes resulted in full wound healing after 36 h, while other nanocarriers led to a complete wound closure after 48 h.

### 4.7. Invasomes

Invasomes are novel liposomal vesicles composed of phospholipids, ethanol, and terpenes or terpene mixtures. The presence of terpenes and ethanol deforms the structure of lipo-vesicles and enhances penetration and permeability of invasomes [69].

Hatem et al. encapsulated α-arbutin and two functional additives (hyaluronic acid and collagen) inside invasomes to enhance the delivery of this active molecule to deeper layers of the skin and create a depot inside the skin [70]. Results from a clinical study showed that α-arbutin-loaded liposomes can successfully reduce the appearance of melasma, the skin’s hyperpigmentation disorder that is challenging to treat.

### 4.8. Ultrasomes

Ultrasomes are a special type of multilamellar liposomes encapsulating endonuclease V, a DNA repair enzyme purified from bacteria *Escherichia coli*. Ceccoli et al. encapsulated T4 endonuclease V, a pyrimidine-dimer-specific DNA repair enzyme, in liposomes showing that this enzyme enhanced DNA repair replication and improved cell survival following UV irradiation [71]. Later on, Yarosh et al. demonstrated that topically applied liposomes encapsulated with T4 endonuclease V (T4N5 liposomes) remove cyclobutane pyrimidine dimers in UV-irradiated cells and reduce UV-induced skin cancer and immunosuppression [72,73]. Clinical studies showed that topical application of T4N5 liposomes in patients with xeroderma pigmentosum reduced the incidence of actinic keratosis by 68% and of basal cell carcinoma by 30% [74]. Wolf et al. found that topical T4N5 liposomes provide strong photoprotection against UV-induced skin damage by prevention of the upregulation of interleukin-10 and TNF-alpha [75,76]. T4N5 liposomes have excellent potential as active ingredients in the formulation of sunscreens and after sun skin repair products.

### 4.9. Marinosomes

Marinosomes are liposomes based on a natural mixture of marine lipids containing a high amount of polyunsaturated fatty acids (PUFA), particularly those of the n-3 series (n-3 PUFA): eicosapentaenoic acid (EPA) and docosahexaenoic acid (DHA) [77]. Nacka et al. prepared liposomes based on a natural marine lipid extract containing a high amount of PUFA (56% of total phospholipids) and α-tocopherol at 5% added to the marine lipid extract to prevent PUFA oxidation [78]. These liposomes, also known as marinosomes, showed a good stability in conditions that mimic those of topical application in terms of pH, temperature, and calcium concentration [77]. Due to high amount of n-3 PUFA marinosomes reduced inflaammatory response by regulating production of pro-inflammatory mediators—prostaglandin E_2_ (PGE_2_) and interleukin 8 (IL-8) [79]. However, currently there is limited evidence on the application of marinosomes in cosmetics and dermatological formulations. Future research should explore the potential use of marinosomes in topical preparations targeting UV-induced oxidative and inflammatory damage of the skin.

## 5. Conclusions

The stratum corneum, the uppermost layer of the skin, acts as a barrier that controls permeation of bioactive molecules included in cosmeceutical and skincare formulations. The current development of different phospholipid-based vesicular systems has not only helped to enhance skin permeation of active compounds but also to improve the solubility and stability of encapsulated active molecules and provide their controlled and sustained release, which leads to improved bioavailability. However, as new active molecules for cosmeceutical applications are constantly being discovered, it is still crucial to understand their skin permeation behaviour in addition to evaluating the encapsulation potential, stability, and effectiveness of the most appropriate nanocarrier for their skin delivery. In modern cosmetic chemistry, phospholipid-based nanocarriers should improve bioavailability of active cosmetic molecules and provide their targeted delivery and extended release.

## Figures and Tables

**Table 1:** Classification of liposomes by structure.

Lamellarity	Number of Layers	Size
Unilamellar vesicles (ULV) Small unilamellar vesicles (SUV) Large unilamellar vesicles (LUV) Giant unilamellar vesicles (GUV)	1	20–100 nm >100 nm >1.0 μm
Oligolamellar vesicles (OLV)	2–5	100 nm–1.0 μm
Multilamellar vesicles (MLV)	≥5	>500 nm
Multivesicular vesicles (MVV)	Several non-concentrically arranged vesicles	>1.0 μm

**Table 2:** Main advantages and disadvantages of liposomes.

Advantages	Disadvantages
Encapsulate hydrophobic and hydrophilic molecules Increased efficacy of active molecule Improved stability of active molecule Biocompatible Biodegradable Non-toxic and non-immunogenic	High production cost Leakage of encapsulated molecule Low solubility Vulnerable to oxidation and hydrolysis Short half-life Osmotically sensitive
